# Choice history biases in dyadic decision making

**DOI:** 10.1038/s41598-025-96182-5

**Published:** 2025-04-03

**Authors:** Ann Huang, Mathis Pink, Viktoria Zemliak, Artur Czeszumski, Peter König

**Affiliations:** 1 Institute of Cognitive Science, University of Osnabrück, Wachsbleiche 27, 49090 Osnabrück, Germany; 2 Social Neuroscience Lab, Institute of Psychology, Polish Academy of Sciences, Warsaw, Poland; 3 Department of Neurophysiology and Pathophysiology, University Medical Center Hamburg-Eppendorf, Hamburg, Germany

**Keywords:** Psychology and behaviour, Perception

## Abstract

How do we interact with our environment and make decisions about the world around us? Empirical research using psychophysical tasks has demonstrated that our perceptual decisions are influenced by past choices, a phenomenon known as the “choice history bias” effect. This decision-making process suggests that the brain adapts to environmental uncertainties based on history. However, single-subject experiment task design is prevalent across the work on choice history bias, thus limiting the implications of the empirical evidence to individual decisions. Here, we explore the choice history bias effect using a dual-participant approach, where dyads perform a shared perceptual decision-making task. We first propose two competing hypotheses: the participants equally weigh their own and their partner’s decision history, or the participants do not weigh equally their own and their partner’s decision history. We then use a statistical modeling approach to fit generalized linear models to the choice data in a series of steps and arrive at a model that best fits the observed data. Our results indicated that the own and partner’s trial history cannot be treated independently. The findings suggest an interaction of actor and decision at 1-back, leading to a choice alternation bias after a partner’s decision in contrast to a choice repetition bias after an own decision. A similar effect is observed at 2-back, in addition to an additive choice repetition bias of similar size. The effects of actor and decision at 2-back do not depend on the properties of the 1-back trial. Together, these findings support the idea that the participants do not ignore their partner’s decisions but treat these qualitatively differently from their own.

**Supplementary Information:**

The online version contains supplementary material available at 10.1038/s41598-025-96182-5.

## Introduction

In daily life, people perceive and process uncertain sensory information to make decisions that lead to useful actions. For example, medical professionals examine X-ray scans to determine signs of abnormalities, or badminton players judge whether the shuttle during a double-player match landed inside or outside the court line. This ability to make perceptual judgments is central to human cognition^1^. In particular, it involves mapping noisy sensory information as input and transforming it into decision responses as output. As such, classical psychophysical methods are often used to describe this perceptual process to understand cognition better^2,3^.

Notably, extensive work on perceptual processing has demonstrated that past choices influence current decisions, a phenomenon referred to as “choice history bias”^4,5^. This effect has been shown using perceptual tasks such as a two-alternative forced-choice (2AFC) task in which participants are asked to discriminate the direction of motion in visual stimuli^6,7^. A choice history bias effect is also found when the stimuli presented on successive trials are uncorrelated^8^. Such empirical evidence suggests the history bias effect persists as a suboptimal decision-making process in which the brain adapts to environmental uncertainties^9^. Therefore, perceptual decisions are influenced by experiment trial history even when the task is not adaptive.

Consistent across the studies on the choice history bias effect is the use of single-subject designs independently of social settings. For instance, Abrahamyan and colleagues examined the adaptability of choice history bias using data collected from individuals across three laboratories^4^. Urai et al. analyzed choice data collected from multiple perceptual experiments across different sensory modalities conducted at the level of single subjects^7^ Nonetheless, in reality, people are not isolated decision-makers. Rather, people often interact with others and integrate existing information available to them, such as when looking at maps together to navigate physical surroundings. In this scenario, one can be influenced by the social cues of others or their own bias in the decision process. Therefore, when standard psychophysical task designs do not account for interaction, insights drawn from these works remain limited to individual decisions.

Research on joint attention implicated the influence of social cues and shared attention on perceptual judgments. For example, Seow and Fleming experimented to test whether perceptual sensitivity depends on social context^10^. By asking participants to detect low-contrast Gabor patches, they discovered that participants’ detection performance improved when the perception was shared with a virtual human, or an avatar. This indicates that individuals consider the visual perspective of others when making perceptual judgments. Experimental work by Wahn and colleagues used joint visual-spatial tasks and linear modeling analyses to investigate how social factors, e.g., information about the co-actor’s actions or performance feedback, might account for group benefits^11,12^. The result of their stepwise modeling approach showed an accurate prediction of collaborative benefits and contributed towards understanding joint action in social cognition. Thus, perception and action are not solely individualistic processes but can be shaped by the dyadic nature of human interactions^13–16^.

Here, we aim to explore how the choice history bias effect might be modulated in a social context. Specifically, we examine the participants’ choice behavior while they take turns performing a shared perceptual task, with stimuli presented in a random sequence, with their dyadic partner. The task allows the participants to observe each other’s choice response on each trial, which could, in turn, influence their decision-making process. This form of interaction in which there is no actual collaboration or feedback has been shown to influence one’s own cognitive processing^17,18^.

Our research objective is to determine whether perceptual decision-making is more of an individualistic (independent of the co-actor’s action) or collective (contingent on the co-actor’s action) process despite the co-actor’s actions being irrelevant to the present decision. For this, we formulate and test competing hypotheses that reflect separate assumptions regarding the choice history bias effect in a social context. The null hypothesis (H0) states that the participants equally weigh their own and their partner’s decision history. This suggests that the choice history bias effect is not limited to a specific actor in the dyad but relates to the combined sequence of decisions by the dyad. The alternative hypothesis (H1) states that the participants do not equally weigh their own and their partner’s decision history. This assumes the choice history effect is influenced by the specific actor. If H1 is supported, we examine how the choice history effect depends on the specific actor. In particular, the participants could either follow or deviate from their partner’s decision. To follow the decision means if the dyadic partner responded left, the participant is likely to choose left, and vice versa for right response (i.e., a “choice repetition”, regression coefficient estimate β > 0). To deviate from the decision means if the dyadic partner responded left, the participant is likely to respond oppositely from this by choosing right, vice versa for right response (i.e., a “choice alternation”, regression coefficient estimate β < 0). We examine the fit of generalized linear models corresponding to the different hypotheses to trial-by-trial choice response in a series of steps. The goal is to arrive at a model that best fits the behavioral data and, in turn, explains the extent to which the hypotheses are supported. Here, we test which hypothesis best fits our observations.

## Materials and methods

### Participants

Seventy-eight individuals, grouped in thirty-nine dyads, were recruited for the present study. Twelve participants (six dyads) were excluded from performing the main experimental task due to exceptionally poor performance during the practice block. This leaves 33 dyads, or 66 individuals (N = 66, 44 females, 21 males, one non-binary, M = 25 years old, SD = 5 years). All participants had normal or corrected-to-normal vision without a history of neurologic or psychiatric illnesses. All participants provided written informed consent before the experiment. The study was conducted in accordance with the Declaration of Helsinki and approved by the Ethics Committee of the University of Osnabrück.

### Experiment protocol

**Fig. 1:**
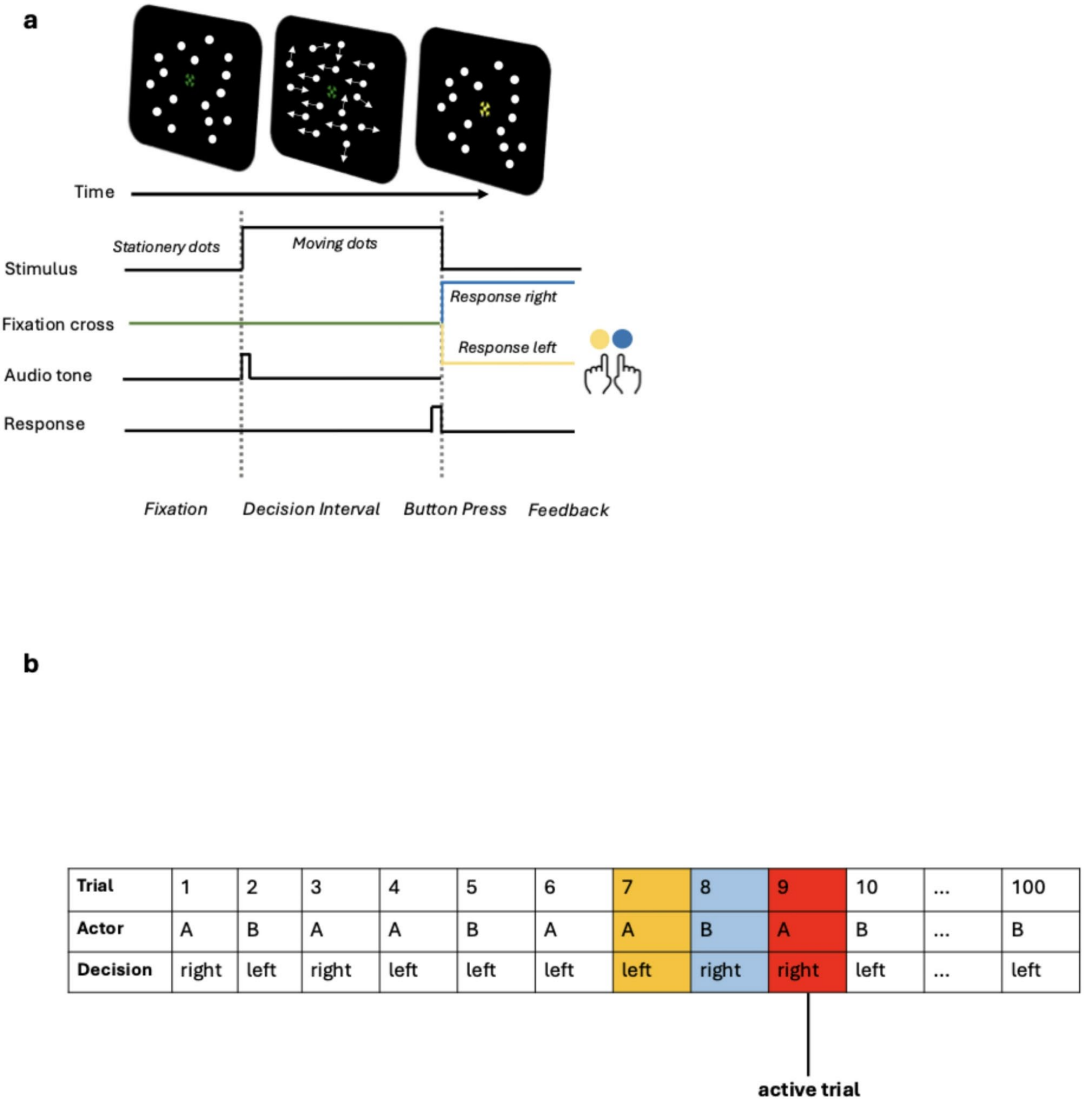
(**a**) A depiction of the experiment task procedure. In each trial, an audio tone cues the participant to respond to the dots that motion dominantly to the left or right. The fixation cross color changed from green to blue or yellow according to the choice response. (**b**) The diagram presents an example sequence of one experiment block performed by one dyad, consisting of participants ”A” and “B.” The choice response, or the decision on each trial, was either right or left. In each trial, either participant A or participant B is the “actor” who performs the task. Here, trial 9, highlighted in red, is shown as an example of an “active trial,” in which participant A is the “acting participant,” while participant B is the “observing participant.” The previous trial with the actor and decision are highlighted in blue. The actor and decision two trials ago are highlighted in yellow. Specifically, trial 8 refers to the trial at “1-back”, in which the observing participant (“partner” or “other”) gave a “right” response. Trial 7 refers to the trial at “2-back”, in which the acting participant (“own” or “self”) gave a “left” response. Overall, the decisions and actors at both delay of one and delay of two from the active trial are depicted.

A speeded random dot motion (RDM) discrimination task was used (Fig. 1a). The task involved viewing a cloud of moving dots and determining whether there was coherent movement rightward or leftward by pressing the two colored buttons (blue = right, yellow = left) on the custom keyboard (Black Box Toolkit USB Response Pads [URP48/URPVK], blackboxtoolkit.com) accordingly. Before the participants began the task, the experimenter gave both written and verbal instructions on the experiment procedure. The experimenter also demonstrated how to make a response by using the keyboard buttons. Participants were instructed to perform the task as quickly and as accurately as possible. In addition, participants were instructed to fixate their eyes on the center of the stimulus presented as a green cross when performing the task.

All stimuli were created in Python (version 3.9.2) using the Psychophysics Toolbox version 2021.1.3^19^. The stimuli’s leftward and rightward movement directions were equiprobable and randomly selected across trials. The dots (N = 328) were white with a size of 3 × 3 pixels, circular aperture of 5° diameter, speed of 9.95°/s, and density of 16.70 dots/degree^2^. They were presented against a black background. The coherence of the stimuli, defined as the proportion of dots moving in the signal direction, was pre-determined. For instance, at a coherence level of 0.5, half of all the dots moved in the trial’s direction, which was set to either 0 or 180 degrees (leftward or rightward) on every frame. These dots constituted the “signal dots’'. The remaining half was referred to as the “noise dots”, where each followed a random but constant direction on each frame. In order to direct the participant’s gaze at the stimuli and keep any involuntary eye movements or drift to a minimum for extended time periods, a bullseye fixation cross was used^20^. For every trial, the fixation cross color changed from green to either blue or yellow for 700 ms post-response to indicate the acting participant’s choice response (yellow if responded ‘left,’ blue if responded ‘right’). During this feedback interval, the dots were stationary. The participant’s partner also saw such feedback information in the main experiment.

In general, our task design and procedure closely replicated established work on perceptual decision-making, particularly that of^6^ where they quantified the decision-making parameters. Our study consisted of two sessions: testing and the main experiment. In the testing session, the participants individually and separately performed the practice block followed by the titration block. The practice block consisted of 40 trials of moving dots at a fixed coherence of 0.4. The titration block consisted of 240 trials with randomly selected dot coherences (0, 0.05, 0.1, 0.2, 0.4, 0.8, 40 trials each). While one participant of a given dyad was performing the practice and titration blocks, the other participant was instructed to wait quietly outside the experiment room. There was a short break between the practice and the titration blocks. If the participants did not achieve an accuracy level of 75% during practice, they were afforded another opportunity (3 practices in total) to repeat the practice block before proceeding to the titration block. In the titration block, the individual coherence threshold was estimated from a psychometric function fit to yield a goal accuracy level of 75%^6^. If the participants failed to reach the goal accuracy or higher, they were excluded from participating in the main experiment, and the experiment was aborted.

The main experiment consisted of 10 blocks with 100 trials each. In the main experiment, the two participants of a dyad sat in separate experimental rooms to perform the task. They alternated randomly to respond to the stimuli presented on a 24-inch-wide Dell U2412M monitor with a resolution of 1920 × 1200 pixels and a refresh rate of 60 Hz at a viewing distance of 60 cm. On each trial, only one dyad member was assigned to respond to the stimulus. Note that while the participants sat in separate rooms, each viewed stimuli moving in an identical direction through an extended window display. However, the stimuli difficulty level was tailored to each participant’s behavioral data, and it was updated after every block (see Supplementary Methods for details on the adaptive procedure). The viewing distance was measured from the participant’s eye to the center of the monitor. The participants self-adjusted the chair’s height to view the center of the stimulus comfortably and placed their index fingers on the custom keyboard to make a response. The chair was fixed to the floor with the help of the experimenter. After every two blocks of the experiment, the experimenter measured the participant’s viewing distance again to keep the viewing distance equal.

At the beginning of the main experiment, the dyads underwent sound familiarization trials. They were trained to recognize their own and their partner’s tones as cues to respond in a given trial. The two distinct tones in the sound familiarization trials were musical notes “C” at octave 5 and “F” at octave 4. Each note had a duration of 0.5 s and was played to the participants 5 times. Lexical instructions accompanied the playing of the tones: “When you hear this, it’s your turn to respond” and “When you hear this, your partner will respond.”

Note that while the testing session consisted of lexical feedback on the response correctness, i.e., a green “Correct” or a red “Incorrect” word was presented below the stimulus after every trial, such feedback was absent during the main experiment. In addition, following the decision interval of 1500 ms after stimulus onset as used in^6^, we also set lexical warnings for response time < 100 ms (“Too Fast”) and > 1500 ms (“Too Slow”). Similarly, during the main experiment, lexical warnings “Partner Too Slow” and “Partner Too Fast” were indicated to the observing participant in the dyad.

After completing the experiment, the participants were asked to complete questionnaires on their demographic data and how well they know their partner on a 100-point scale. The testing session for each participant lasted about 30 min. The main experiment took around 2 h; therefore, the entire experiment took about 3 h for each pair of participants. Overall, the experiment set-up followed closely past empirical work, where the interaction between the dyads is solely within the perceptual task.

### Method of data analysis

#### Terminologies and variable coding

Here, we describe the variable names and terminologies used throughout the study. Overall, the objective of creating the following variables was to build a model that could first capture any linear effect on the current choice up to the delay of two. This allowed an unbiased data-driven investigation of the choice history bias in dyadic decision-making. In discussing the details of the current trial, the variable $S_{n}$ coded for the stimulus; furthermore, the variable $A_{n}$ coded for the identity of the participant acting, while the variable $D_{n}$ coded for the decision, or choice response. As illustrated in Fig. 1b, the actors and decisions at 1-back and 2-back are in relation to the active trial. In discussing the trial at 1-back, the variables $A_{n - 1}$ and $D_{n - 1}$ coded for information about the previous trial actor identity and decision, respectively. Similarly, for the trial at 2-back, the variables $A_{n - 2}$ and $D_{n - 2}$ coded for information about the identity of the actor and decision at two trials ago, respectively. + 1 and − 1 were used in the coding of the decision (+ 1 = right; − 1 = left), stimulus (+ 1 = right; − 1 = left), and actor identities (+ 1 = own; − 1 = “partner”).

In discussing trial history up to the delay of two, we combined the actor and decision at 2-back with the details of the actor and decision at 1-back. Specifically, we created variables containing such information using conditional effect coding, or “one hot encoding”. + 1, − 1 and 0 were used to indicate the categories of the conditions met. For example, the variable $(A_{n - 1}^{ + 1} )(D_{n - 1}^{ - 1} )*\left( {A_{n - 2} } \right)$ codes for the actor at 2-back, but only under the condition that 1-back was performed by “own” with decision “left”. Therefore, $(A_{n - 1}^{ + 1} )(D_{n - 1}^{ - 1} )*\left( {A_{n - 2} } \right)$ is + 1 indicates “own” at 2-back, and − 1 indicates partner at 2-back, conditioned on the fact that 1-back was performed by “own” with decision “left”. If the 1-back condition of “own” and decision “left” is not met, the variable $(A_{n - 1}^{ + 1} )(D_{n - 1}^{ - 1} )*\left( {A_{n - 2} } \right)$ is 0. Similarly, the variable $(A_{n - 1}^{ + 1} )(D_{n - 1}^{ - 1} )*\left( {D_{n - 2} } \right)$ codes for the decision at 2-back, but only under the condition that 1-back was performed by “own” with a decision “left”. As such, $(A_{n - 1}^{ + 1} )(D_{n - 1}^{ - 1} )*\left( {D_{n - 2} } \right)$ is + 1 indicates decision “left” at 2-back, and -1 indicates decision “right” at 2-back, conditioned on the fact that 1-back was “own” and “left”. If the 1-back conditions of “own” and decision “left” was not met, $(A_{n - 1}^{ + 1} )(D_{n - 1}^{ - 1} )*\left( {D_{n - 2} } \right)$ is 0. Table 1 presents the variable names and descriptions until 2-back. Note that we did not create variables that coded the combination of actor and their directional decision at 1-back (e.g., 1-back performed by own with left decision), resulting in a total of 4 combinations. Furthermore, we did not create variables that combined the actor identity and directional decision at 1-back with those at 2-back (e.g., 1-back performed by own with left decision, combined with 2-back performed by own with left decision), resulting in a total of 16 combinations. This is because the use of variables coded as such leads to multicollinearity which introduces complexities in interpretations. For logistic regression modeling estimation, the dependent variable is the participants’ response choices, which were bounded between 0 and 1, reflecting the probability of selecting rightward, rising from 0 (left) to 1 (right).

**Table 1:** Variable names and the descriptions of what they code for.

Variable name	Description
$S_{n}$	The current trial stimulus
$A_{n}$	The current trial actor
$D_{n}$	The current decision or choice response
$A_{n - 1}$	Actor at 1-back
$D_{n - 1}$	Decision at 1-back
$A_{n - 2}$	Actor at 2-back
$D_{n - 2}$	Decision at 2-back
$(A_{n - 1}^{ + 1} )\left( {D_{n - 1}^{ - 1} } \right)*\left( {A_{n - 2} } \right)$	Actor at 2-back, under the condition that 1-back was “own” and decision “left”
$(A_{n - 1}^{ + 1} )\left( {D_{n - 1}^{ - 1} } \right)*\left( {D_{n - 2} } \right)$	Decision at 2-back, under the condition that 1-back was “own” and decision “left”
$(A_{n - 1}^{ + 1} )\left( {D_{n - 1}^{ + 1} } \right)*\left( {A_{n - 2} } \right)$	Actor at 2-back under the condition that 1-back was “own” and decision “right”
$(A_{n - 1}^{ + 1} )\left( {D_{n - 1}^{ + 1} } \right)*\left( {D_{n - 2} } \right)$	Decision at 2-back, under the condition that 1-back was “own” and decision “right”
$(A_{n - 1}^{ - 1} )\left( {D_{n - 1}^{ - 1} } \right)*\left( {A_{n - 2} } \right)$	Actor at 2-back, under the condition that 1-back was “partner” and decision “left”
$(A_{n - 1}^{ - 1} )\left( {D_{n - 1}^{ - 1} } \right)*\left( {D_{n - 2} } \right)$	Decision at 2-back, under the condition that 1-back was “partner” and decision “left”
$(A_{n - 1}^{ - 1} )\left( {D_{n - 1}^{ + 1} } \right)*\left( {A_{n - 2} } \right)$	Actor at 2-back, under the condition that 1-back was “partner” and decision “right”
$(A_{n - 1}^{ - 1} )\left( {D_{n - 1}^{ + 1} } \right)*\left( {D_{n - 2} } \right)$	Decision at 2-back, under the condition that 1-back was “partner” and decision “right”

The table shows the coding of the current trial stimulus and decision, followed by the actors and decisions up to the delay of two. The letters “S”, “A” and “D” stand for “stimulus”, “actor” and “decision”, respectively. The asterisk indicates the conditioning between two factors.

#### Generalized linear modeling

To test our hypotheses, we fitted generalized linear models (GLMs) with logit link function to quantify the influence of trial history on choice behavior. Following prior work on modeling choice history biases^7,21^, we used the Akaike Information Criterion (AIC) values^22^ for formal model comparisons. Given the large number of trials, we also used the Bayesian Information Criterion (BIC) values, which penalizes model complexity more strongly than AIC^23^. To assess the model performance, we conducted cross-validation by partitioning the data into training and testing sets. We evaluated the models using cross-validated accuracy and mean squared error (MSE). The binomial logistic regression estimated the probability of selecting the right decision response based on the weighting of both sensory (i.e., current stimulus) and nonsensory parameters (e.g., past trial responses). The model distinguished response biases, such as when the participants preferred to repeat or switch their choice response. We chose to use effect coding for easier interpretation of the coefficient estimates as they directly indicate the difference in the mean outcome variable between the two levels of the predictor variables.

## Results

### Exploratory data analysis

**Fig. 2:**
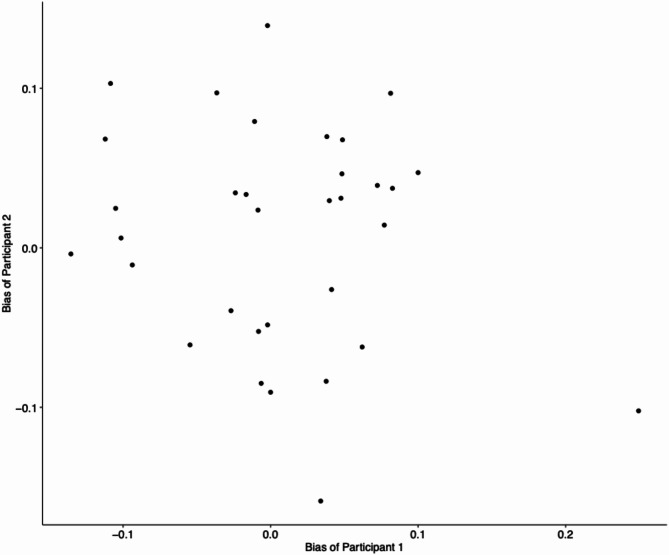
A scatterplot of the left/right bias values of two participants within the same dyad when giving a response. Each dot represents a dyad, with the bias value of one participant shown on the x-axis and the bias value of the other participant shown on the y-axis. Pearson correlation showed no significant correlation or anticorrelation in the observed pattern.

First, we processed and examined the recorded responses in the main experiment to check for any missing responses and potential left/right bias in the participants. In total 33,000 responses were recorded, i.e., no missing responses observed. Following this, only trials with reaction time (RT) greater than 0.1 s and less than 1.5 s were included in the subsequent analysis, resulting in a removal of 1795 trials out of 33,000 trials (5.44% of total trials). The number of active trials per participant ranged from 338 to 523, with a mean of 472 trials (SD = 30). One dyad had a difference of 129 trials in terms of the number of active trials. The average number of right responses per participant was 240 (range = 161–350, SD = 39), while the average number of left responses was 232 (range = 107–324, SD = 39). On the dyad level, the average number of left responses was 465 (range = 348–545, SD = 54.57), and the average number of right responses was 480 (range = 410–581, SD = 45). To examine any left/right bias i.e., a preference to favor one response over another, we calculated the difference between the proportion of right choice responses and the proportion of rightward-moving stimulus for the two participants within the same dyad. A resulting positive value indicates a tendency to respond right, and negative value indicates a tendency to respond left. The mean bias values across the participants is 0.007 (range = − 0.159–0.249, SD = 0.073). Figure 2 shows a visualization of the bias values between the two participants of the same dyad. Pearson’s correlation between the bias values of the two participants within the dyads showed no significant correlation or anti-correlation, which allows for subsequent investigation on the influence of choice history bias without adjustments for potential effect of left-biased participant pairing with right-biased participant, or vice versa.

**Fig. 3:**
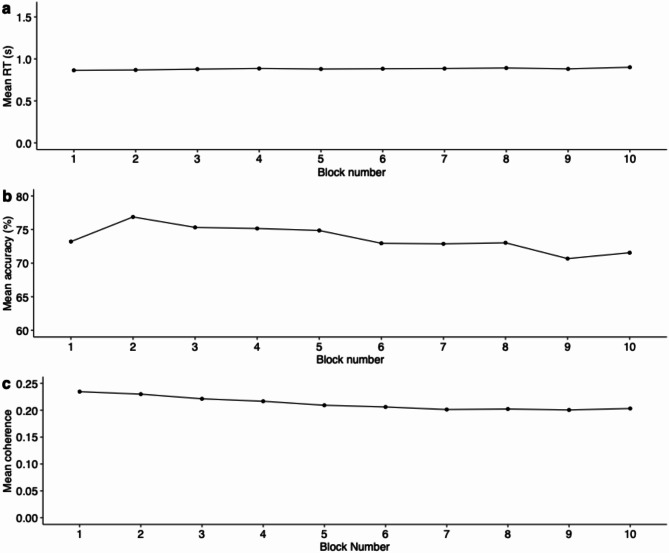
Changes in the major dependent variables throughout the main experiment. (**a**) The RT across the experiment blocks. (**b**) The mean accuracy performance throughout the experiment. (**c**) The main experiment’s mean coherence level (updated after every block for each participant).

We analyzed the RT, performance accuracy, and coherence level data to check for variations across the blocks. The average RT was 0.87 s (SD = 0.25 s), and the average performance accuracy was 73.6% (SD = 5.87%). The individual coherence threshold level range was 0.20–0.23, with a mean of 0.21 (SD = 0.012). Figure 3 shows the participant’s mean RT, accuracy, and adapted coherence level changes throughout the main experiment. Pearson’s correlation between the accuracy and stimuli coherence shows a significant positive but weak correlation (r = 0.12, *p* < 0.001). The correlation between RT and accuracy was negative and significant (r = − 0.23, *p* < 0.001); furthermore, the correlation between coherence and RT was negative and significant (r = − 0.18, *p* < 0.001). The consistency in the mean RT and performance with a slightly decreasing trend for stimuli coherence suggested the adaptive procedure worked reasonably well.

### Modeling choice history biases

The modeling approach began with a no-history baseline model, followed by incorporating trial actors and choice history data up to a delay of two. Building on past empirical work that showed previous decisions influenced the participant’s choice in the absence of single-trial feedback and irrespective of previous choice correctness^7^, we assumed that each dyadic participant viewed their decisions as correct. This modeling logic avoided introducing qualitatively different models that could e.g., account for the participant’s perceived correctness for both their own and their partner’s decisions, which kept the modeling approach simple and consistent with past conventions. We assessed the models in relation to the hypotheses, with an objective to arrive at one arguably less complex model that is simpler to interpret and assumed the best fit. In the following, we report the results of each modeling step.

First, we examined a task-only model, where only the current trial stimulus was included as a predictor to model the participants’ performance without any additional constraints, such as what the previous response was or the identity of the previous trial actor:

$$Pr\left( {D_{n} = 1} \right) \sim S_{n}$$

This Model 0 served as a baseline model and assumed the estimation of the current response depends solely on the current trial stimulus, which was the actual task. The results showed a statistically significant effect of the current stimulus on the current response (β = 1.04, 95% CI [1.01, 1.06], SE = 0.03, *p* < 0.001). Standard errors were clustered at the participant level to account for the non-independence of observations within individuals. The cross-validated accuracy of the model’s prediction was 73.8%, with an MSE of 0.1933. The accuracy value was computed by comparing the model’s predicted probabilities (in values 0 s and 1 s) against the true labels of the outcome variable. The MSE value was calculated as the average squared difference between the predicted and the true values. The threshold for transforming the predicted probabilities into predicted labels was set at 0.5. If the predicted probability for a rightward response exceeded 0.5, we interpreted it as a prediction for class 1 (rightward response). Otherwise, the predicted probabilities below 0.5 were classified as a prediction for class 0 (leftward response). The predicted probability for a rightward response given a rightward-moving stimulus derived from the model’s estimates was 73.8%, which aligned with our intention of the task design to yield a goal of about 75% accuracy performance. The significant and positive association between the predictor and the response suggested the participants followed task instructions and behaved as they should.

To investigate how trial history could influence the participant’s choice, we built on the baseline model to include the variables that accounted for the possible sequences of actors and the decisions up to the delay of two:

$$\begin{aligned} Pr\left( {D_{n} = 1} \right) & \sim S_{n} + A_{n - 1} + D_{n - 1} + A_{n - 1} \times D_{n - 1} \\ & \quad + (A_{n - 1}^{ + 1} )\left( {D_{n - 1}^{ - 1} } \right)*\left( {A_{n - 2} } \right) + (A_{n - 1}^{ + 1} )\left( {D_{n - 1}^{ - 1} } \right)*\left( {D_{n - 2} } \right) \\ & \quad + (A_{n - 1}^{ + 1} )\left( {D_{n - 1}^{ - 1} } \right)*\left( {A_{n - 2} } \right) \times (A_{n - 1}^{ + 1} )\left( {D_{n - 1}^{ - 1} } \right)*\left( {D_{n - 2} } \right) \\ & \quad + (A_{n - 1}^{ + 1} )\left( {D_{n - 1}^{ + 1} } \right)*\left( {A_{n - 2} } \right) + (A_{n - 1}^{ + 1} )\left( {D_{n - 1}^{ + 1} } \right)*\left( {D_{n - 2} } \right) \\ & \quad + (A_{n - 1}^{ + 1} )\left( {D_{n - 1}^{ + 1} } \right)*\left( {A_{n - 2} } \right) \times (A_{n - 1}^{ + 1} )\left( {D_{n - 1}^{ + 1} } \right)*\left( {D_{n - 2} } \right) \\ & \quad + (A_{n - 1}^{ - 1} )\left( {D_{n - 1}^{ - 1} } \right)*\left( {A_{n - 2} } \right) + (A_{n - 1}^{ - 1} )\left( {D_{n - 1}^{ - 1} } \right)*\left( {D_{n - 2} } \right) \\ & \quad + (A_{n - 1}^{ - 1} )\left( {D_{n - 1}^{ - 1} } \right)*\left( {A_{n - 2} } \right) \times (A_{n - 1}^{ - 1} )\left( {D_{n - 1}^{ - 1} } \right)*\left( {D_{n - 2} } \right) \\ & \quad + (A_{n - 1}^{ - 1} )\left( {D_{n - 1}^{ + 1} } \right)*\left( {A_{n - 2} } \right) + (A_{n - 1}^{ - 1} )\left( {D_{n - 1}^{ + 1} } \right)*\left( {D_{n - 2} } \right) \\ & \quad (A_{n - 1}^{ - 1} )\left( {D_{n - 1}^{ + 1} } \right)*\left( {A_{n - 2} } \right) \times (A_{n - 1}^{ - 1} )\left( {D_{n - 1}^{ + 1} } \right)*\left( {D_{n - 2} } \right) \\ \end{aligned}$$

This Model 1 included the trial history up to 2-back and accounted for the complete combinations of the sequences of past actors and choices, i.e., whether the preceding 1- and 2-back trials were performed by oneself or the other giving a right or a left response when predicting the choice to be made. The results showed significant and positive main effects for the variables $S_{n}$ (β = 1.04, 95% CI [1.02, 1.07], SE = 0.03, *p* < 0.001), $(A_{n - 1}^{ + 1} )\left( {D_{n - 1}^{ - 1} } \right)*\left( {D_{n - 2} } \right)$(β = 0.08, 95% CI [0.01, 0.15], SE = 0.04, *p* < 0.05) and $(A_{n - 1}^{ + 1} )\left( {D_{n - 1}^{ + 1} } \right)*\left( {D_{n - 2} } \right)$(β = 0.08, 95% CI [0.01, 0.14], SE = 0.03, *p* < 0.05). The model also showed all the interaction variables positive and significant: $A_{n - 1} \times D_{n - 1}$(β = 0.11, 95% CI [0.07, 0.14], SE = 0.02, *p* < 0.001), $(A_{n - 1}^{ + 1} )\left( {D_{n - 1}^{ - 1} } \right)*\left( {A_{n - 2} } \right) \times (A_{n - 1}^{ + 1} )\left( {D_{n - 1}^{ - 1} } \right)*\left( {D_{n - 2} } \right)$ (β = 0.27, 95%, CI [0.17, 0.38], SE = 0.06, *p* < 0.001), $(A_{n - 1}^{ + 1} )\left( {D_{n - 1}^{ + 1} } \right)*\left( {A_{n - 2} } \right) \times (A_{n - 1}^{ + 1} )\left( {D_{n - 1}^{ + 1} } \right)*\left( {D_{n - 2} } \right)$ (β = 0.22, 95% CI [0.12, 0.32], SE = 0.06, *p* < 0.001), $(A_{n - 1}^{ - 1} )\left( {D_{n - 1}^{ - 1} } \right)*\left( {A_{n - 2} } \right) \times (A_{n - 1}^{ - 1} )\left( {D_{n - 1}^{ - 1} } \right)*\left( {D_{n - 2} } \right)$ (β = 0.19, 95% CI [0.09, 0.30], SE = 0.07, *p* < 0.001), and $(A_{n - 1}^{ - 1} )\left( {D_{n - 1}^{ + 1} } \right)*\left( {A_{n - 2} } \right) \times (A_{n - 1}^{ - 1} )\left( {D_{n - 1}^{ + 1} } \right)*\left( {D_{n - 2} } \right)$ (β = 0.14, 95% CI [0.04, 0.24], SE = 0.06, *p* < 0.01). The model exhibited an AIC value of 31,314.15 (Δ_AIC_ =  − 139 units from Model 0), as well as a BIC value of 31,453.83 (Δ_BIC_ =  − 15.72 units from the Model 0). The cross-validated accuracy of the model was 73.8%, with an MSE value of 0.1923. Note, however, that the accuracy value was computed based on predictions thresholded at 0.5, thus not fully reflecting the variations in the model’s factual prediction values. Overall, Model 1 demonstrates the impact of past decisions and actors that bias the decision-making.

As a next step, we investigated whether the history of own and partner’s decisions can be considered independently. That is, does it matter whether the last own decision occurred directly before or after the last partner’s decision? Specifically, we calculated the predicted influence based on the specific combination of actor sequences to determine if the order of own or partner action matters. In other words, given the estimates from Model 1 we computed the predictions for each specific case and checked the effect of actor and directional decisions. For example, $(A_{n - 1}^{ + 1} D_{n - 1}^{ - 1} )\left( {A_{n - 2}^{ - 1} D_{n - 2}^{ - 1} } \right)$ is own left decision at 1-back, combined with partner left decision at 2-back, which is calculated by the following: (β of $A_{n - 1}$)(own) + (β of $D_{n - 1}$)(own at 1-back)(decision left at 1-back) + (β of $(A_{n - 1}^{ + 1} )\left( {D_{n - 1}^{ - 1} } \right) *\left( {A_{n - 2} } \right)$)(partner at 2-back) + (β of $(A_{n - 1}^{ + 1} )\left( {D_{n - 1}^{ - 1} } \right)*\left( {D_{n - 2} } \right)$(decision left at 2-back) + (β of $(A_{n - 1}^{ + 1} )\left( {D_{n - 1}^{ - 1} } \right)*\left( {A_{n - 2}^{{}} } \right) \times (A_{n - 1}^{ + 1} )\left( {D_{n - 1}^{ - 1} } \right)*\left( {D_{n - 2}^{{}} } \right)$(partner at 2-back x decision left at 2-back). This is compared with $(A_{n - 1}^{ - 1} D_{n - 1}^{ - 1} )\left( {A_{n - 2}^{ + 1} D_{n - 2}^{ - 1} } \right)$, which is partner left decision at 1-back followed by own left decision at 2-back, and is calculated by the following: (β of $A_{n - 1}$)(partner) + (β of $D_{n - 1}$)(partner at 1-back)(decision left at 1-back) + (β of $(A_{n - 1}^{ - 1} )\left( {D_{n - 1}^{ - 1} } \right) *\left( {A_{n - 2} } \right)$)(own at 2-back) + (β of $(A_{n - 1}^{ - 1} )\left( {D_{n - 1}^{ - 1} } \right)*\left( {D_{n - 2} } \right)$(decision left at 2-back) + (β of $(A_{n - 1}^{ - 1} )\left( {D_{n - 1}^{ - 1} } \right)*\left( {A_{n - 2} } \right) \times (A_{n - 1}^{ - 1} )\left( {D_{n - 1}^{ - 1} } \right)*\left( {D_{n - 2} } \right)$(own at 2-back x decision left at 2-back). Accounting for the trials up to the delay of two i.e., 1-back and 2-back, this makes a total of 4 directional decision combinations: 1) left/left, 2) right/right, 3) left/right, and 4) right/left. For each of the four cases, we calculated and compared the results when swapping the actor order (own vs. partner). The details of the manual calculations are reported in the Supplementary Results. Overall, we observe deviant results for the estimate in the current trial depending on whether the own or partner’s last decision occurred earlier (Case 1: 0.08 vs -0.12; Case 2: -0.04 vs. 0.03; Case 3: -0.30 vs. 0.32; Case 4: 0.30 vs. -0.29). Additionally, swapping the actor’s order in each decision case shows sign flips. Thus, the history of one’s own decisions and the history of partner’s decisions cannot be treated independently, rather, the own and partner’s trials with how various decisions interdigitate have to be considered.

Based on the previous results, we turn our attention to potential simplifications of the model related to the dependencies of trials at 1-back and 2-back. Specifically, we tested whether the actor and the directional decision at 2-back are independent of the directional decision at 1-back. For this, we developed Model 2 which is simplified:

$$\begin{aligned} Pr\left( {D_{n} = 1} \right) & \sim S_{n} + A_{n - 1} \times D_{n - 1} \\ & \quad + (A_{n - 1}^{ + 1} )*(A_{n - 2}^{{}} ) + (A_{n - 1}^{ + 1} )*(D_{n - 2}^{{}} ) \\ & \quad + (A_{n - 1}^{ + 1} )*(A_{n - 2}^{{}} ) \times (A_{n - 1}^{ + 1} )*(D_{n - 2}^{{}} ) \\ & \quad + (A_{n - 1}^{ - 1} )*(A_{n - 2}^{{}} ) + (A_{n - 1}^{ - 1} )*(D_{n - 2}^{{}} ) \\ & \quad + (A_{n - 1}^{ - 1} )*(A_{n - 2}^{{}} ) \times (A_{n - 1}^{ - 1} )*(D_{n - 2}^{{}} ) \\ \end{aligned}$$

The variables incorporated assume the actor or decision at 2-back is conditional on the actor at 1-back but do not depend on the decision at 1-back. The results showed significant and positive main effects for the variables $S_{n}$ (β = 1.04, 95% CI [1.02, 1.07], SE = 0.03, *p* < 0.001), $(A_{n - 1}^{ + 1} )*(D_{n - 2}^{{}} )$(β = 0.11, 95% CI [0.17, 0.16], SE = 0.02, *p* < 0.001), and $(A_{n - 1}^{ - 1} )*(D_{n - 2}^{{}} )$ (β = 0.07, 95% CI [0.02, 0.11], SE = 0.03, p < 0.01). For the interaction variables, $A_{n - 1} \times D_{n - 1}$ showed positive and significant (β = 0.11, 95% CI [0.09, 0.14], SE = 0.02, *p* < 0.001). Both $(A_{n - 1}^{ + 1} )*(A_{n - 2} ) \times (A_{n - 1}^{ + 1} )*(D_{n - 2} )$ and $(A_{n - 1}^{ - 1} )*(A_{n - 2} ) \times (A_{n - 1}^{ - 1} )*(D_{n - 2} )$ showed very similar positive and significant estimates (β = 0.15, 95%, CI [0.09, 0.22], SE = 0.04, *p* < 0.001 and β = 0.15, 95% CI [0.09, 0.21], SE = 0.04, *p* < 0.001, respectively). The model’s predicted values, i.e., estimated marginal means for the variable $(A_{n - 1}^{ + 1} )*(A_{n - 2} ) \times (A_{n - 1}^{ + 1} )*(D_{n - 2} )$ showed that under the condition that 1-back was own, when the decision at 2-back was right and the actor at 2-back was own, the likelihood of repeating the decision was 0.81, as compared to a lower likelihood of 0.75 when the actor at 2-back was partner. Similarly, the model’s predicted values for the variable $(A_{n - 1}^{ - 1} )*(A_{n - 2} ) \times (A_{n - 1}^{ - 1} )*(D_{n - 2} )$ showed that under the condition that 1-back was partner, when the decision at 2-back was right and the actor at 2-back was own, the likelihood of repeating the decision was 0.80, compared to a lower likelihood of 0.74 when the actor at 2-back was the partner. This indicates that, whether 1-back was own or partner, the participant exhibited a choice repetition bias when their self acted at 2-back. Lastly, Model 2 exhibited an AIC value of 31,294 (Δ_AIC_ = -20.15 units from Model 1), as well as a BIC value of 31,368.08 (Δ_BIC_ = − 85.75 units from the Model 1). The cross-validated accuracy of the model was 73.8%, with an MSE value of 0.1921. This suggests the quantitative effect of the actor at 2-back does not depend on the directional decision at 1-back.

Next, we further simplified the model to test whether the effect of the actor at 2-back is independent of the actor and directional decisions at 1-back:

$$\begin{aligned} Pr\left( {D_{n} = 1} \right) & \sim S_{n} + A_{n - 1} + D_{n - 1} + A_{n - 1} \times D_{n - 1} \\ & \quad + A_{n - 2} + D_{n - 2 } + A_{n - 2} \times D_{n - 2 } \\ \end{aligned}$$

This Model 3 reflects that while the actor and decision at 1-back and 2-back are relevant, the influence of 2-back actors and decisions is not directly dependent on the 1-back actor and decision. The results showed positive and significant effect of $S_{n}$ (β = 1.05, 95% CI [1.02, 1.07], SE = 0.03, *p* < 0.001) and $D_{n - 2 }$(β = 0.13, 95% CI [1.10, 1.16], SE = 0.02, *p* < 0.001). The results also showed positive and significant effects for the interaction $A_{n - 1} \times D_{n - 1 }$(β = 0.11, 95% CI [0.08, 0.14], SE = 0.02, *p* < 0.001) and $A_{n - 2} \times D_{n - 2}$(β = 0.12, 95% CI [0.09, 0.14], SE = 0.02, *p* < 0.001). This model exhibited an AIC of 31,240 (Δ_AIC_ = − 54.13 units from the Model 2) and a BIC of 31,305.46 (Δ_BIC_ = − 62.62 units from Model 1). The cross-validated accuracy value of the model was 73.8%, with a MSE value of 0.1917. Table 2 presents a summary of the regression models. Together, the further implications revealed that Model 3 better fits the observed data. Notably, this model, which does not accommodate any dependence of the effect of 2-back on the details of what happened at 1-back, indicated significant interaction effects of the actors and decisions for both 1-back and 2-back. This suggests the decision and actor jointly influence the choice to be made, showing support for H1. For example, the likelihood of repeating a choice is influenced by the acting participant’s response at 2-back, with the likelihood increasing or decreasing depending on whether the acting participant responded left or right.

**Table 2:** Summary of regression modeling steps.

Predictors	Model 0			Model 1			Model 2			Model 3		
	Log-odds	SE	CI	Log-odds	SE	CI	Lod-odds	SE	CI	Log-odds	SE	CI
(Intercept)	0.04**	0.04	0.02–0.07	− 0.01	0.04	− 0.04–0.02	− 0.00	0.04	− 0.03–0.03	− 0.01	0.04	0.02–0.07
$S_{n}$	1.04***	0.03	1.01–1.06	1.04***	0.03	1.02–1.07	1.04***	0.03	1.02–1.07	1.05***	0.03	1.02–1.07
$A_{n - 1}$				− 0.03	0.02	− 0.07–0.01				− 0.00	0.01	− 0.03–0.02
$D_{n - 1}$				0.00	0.03	− 0.03–0.04				− 0.01	0.02	− 0.03–0.02
$A_{n - 1} \times D_{n - 1}$				0.11***	0.02	0.07–0.14	0.11***	0.02	0.07–0.14	0.11***	0.02	0.08–0.14
$A_{n - 2}$										0.02	0.01	− 0.01–0.04
$D_{n - 2}$										0.13***	0.02	0.10–0.16
$A_{n - 2} \times D_{n - 2}$										0.12***	0.02	0.09–0.14
$(A_{n - 1}^{ + 1} )*(A_{n - 2} )$							0.00	0.02	− 0.04–0.05			
$(A_{n - 1}^{ + 1} )*(D_{n - 2} )$							0.11***	0.02	0.07–0.16			
$(A_{n - 1}^{ + 1} )*(A_{n - 2} )$ × $(A_{n - 1}^{ + 1} )*(D_{n - 2} )$							0.15***	0.04	0.09–0.22			
$(A_{n - 1}^{ - 1} )*(A_{n - 2} )$							0.01	0.02	− 0.03–0.06			
$(A_{n - 1}^{ - 1} )*(D_{n - 2} )$							0.07	0.03	0.02–0.11			
$(A_{n - 1}^{ - 1} )*(A_{n - 2} )$ × $(A_{n - 1}^{ - 1} )*(D_{n - 2} )$							0.15***	0.04	0.09–0.21			
$(A_{n - 1}^{ + 1} )(D_{n - 1}^{ - 1} )*(A_{n - 2} )$				− 0.03	0.04	− 0.09–0.04						
$(A_{n - 1}^{ + 1} )(D_{n - 1}^{ - 1} )*(D_{n - 2} )$				0.08*	0.04	0.01–0.15						
$(A_{n - 1}^{ + 1} )(D_{n - 1}^{ + 1} )*(A_{n - 2} )$				− 0.02	0.03	− 0.08–0.05						
$(A_{n - 1}^{ + 1} )(D_{n - 1}^{ + 1} )*(D_{n - 2} )$				0.08*	0.03	0.01–0.14						
$(A_{n - 1}^{ - 1} )(D_{n - 1}^{ - 1} )*(A_{n - 2} )$				− 0.04	0.03	− 0.10–0.03						
$(A_{n - 1}^{ - 1} )(D_{n - 1}^{ - 1} )*(D_{n - 2} )$				0.03	0.03	− 0.03–0.10						
$(A_{n - 1}^{ - 1} )(D_{n - 1}^{ + 1} )*(A_{n - 2} )$				− 0.05	0.03	− 0.12–0.01						
$(A_{n - 1}^{ - 1} )(D_{n - 1}^{ + 1} )*(D_{n - 2} )$				0.02	0.02	− 0.05–0.09						
$(A_{n - 1}^{ + 1} )(D_{n - 1}^{ - 1} )*(A_{n - 2} )$ × $(A_{n - 1}^{ + 1} )(D_{n - 1}^{ - 1} )*(D_{n - 2} )$				0.27***	0.06	0.17–0.38						
$(A_{n - 1}^{ + 1} )(D_{n - 1}^{ + 1} )*(A_{n - 2} )$ × $(A_{n - 1}^{ + 1} )(D_{n - 1}^{ + 1} )*(D_{n - 2} )$				0.22***	0.06	0.12–0.32						
$(A_{n - 1}^{ - 1} )(D_{n - 1}^{ - 1} )*(A_{n - 2} )$ × $(A_{n - 1}^{ - 1} )(D_{n - 1}^{ - 1} )*(D_{n - 2} )$				0.19***	0.07	0.09–0.30						
$(A_{n - 1}^{ - 1} )(D_{n - 1}^{ + 1} )*(A_{n - 2} )$ × $(A_{n - 1}^{ - 1} )(D_{n - 1}^{ + 1} )*(D_{n - 2} )$				0.14**	0.06	0.04–0.24						
AIC	31,453.117			31,314.154			31,294.131			31,240		
BIC	31,469.55			31,453.83			31,368.08			31,305.46		
Accuracy	0.738			0.738			0.738			0.738		
MSE	0.1932			0.1923			0.1921			0.1917		

The table presents coefficient estimates with 95% confidence intervals (CI) for Model 0, Model 1, Model 2, and Model 3. Standard errors are clustered at the individual level. In addition, the AIC, BIC, cross-validated accuracy, and MSE values are provided for each model.

*p < 0.05 **p < 0.01 ***p < 0.001.

## Discussion

In the present study, we investigated the choice history bias effect in a social context. Specifically, participants were grouped in dyads to perform a shared perceptual decision-making task that allowed each participant to observe their partner’s responses. Using a stepwise regression approach, we tested the extent to which the modeling results support our two proposed hypotheses. Comparisons between the models led to Model 3 as the better-fitting model, which specifies an effect of the task stimulus, the combined influence of the actor and decision at 1-back, the decision at 2-back, and the combined influence of the actor and decision at 2-back. Specifically, the model exhibited lowest AIC and BIC, which indicates a better balance between the model fit and complexity. The reduction in the cross-validated MSE also suggests a decrease in prediction error. Thus, while the predictive accuracy apparently remained constant, likely due to the classification thresholding at 0.5 not reflecting the full variations in the model’s factual prediction values, the improvements in AIC, BIC, and MSE indicate that the model better fits the data. Overall, our findings indicate a dyadic dependency in which the participant did not ignore their partner’s decisions; yet, they treated their partner’s decisions differently from their own i.e., deviated from their partner’s decision.

The current study is exploratory and has limitations. One limitation is that the participants sat in separate experimental rooms and did not share the same peripersonal space. This physical separation may have limited the interaction effect typically accounted for in a shared space^15^. The participants did not communicate but only observed each other’s responses on each trial. While this factor was part of the experiment design, it could reduce a sense of social presence that influences perceptual judgments^24^. On the flip side, these design choices allowed a clean and unambiguous analysis of the dyadic decision process.

Model 3 revealed a complex dyadic dependency on choice behavior influenced by own and partner’s decision history. At 1-back, the variables $A_{n - 1}$ and $D_{n - 1}$ did not show main effects, yet their interaction $A_{n - 1} \times D_{n - 1}$(β = 0.11) does. Interestingly, the model’s predicted values computed for $A_{n - 1} \times D_{n - 1}$ revealed an effect that is opposite for own vs. partner. For instance, when own responds right at 1-back, the predicted value is 0.77, and when partner responds left at 1-back, the value is also 0.77. Similarly, when own responds left at 1-back, the predicted value is 0.73, which is identical to when partner responds right at 1-back (0.73). This indicates that the participant is not ignoring the partner’s previous response but not necessarily adhering to it either. Otherwise, the estimated probability for the choice response, e.g., after the partner responded left at 1-back, would have differed or been lower. Thus, the participant seemed to show some adaptability in response to the partner’s decisions, implicating a degree of social sensitivity. This aligns with past empirical work demonstrating how people can be sensitive to others’ attentional focus in shared tasks^13^. Furthermore, previous research by Bahrami et al. also suggested that the lack of communication as well as feedback between pairs of participants could result in no build-up of collective benefit^24^, which could explain the weak dyadic effect observed here. The results at 1-back implicate a nuance yet limited role in influencing choice behavior.

Building on the discussion of results at 1-back, the model also showed how choice behavior is influenced by own and partner decisions at 2-back. The variable $D_{n - 2 }$(β = 0.13) showed a significant main effect, which is in contrast to the variable $D_{n - 1 }$ that only shows an influence dependent on the actor. Here the decision at 2-back predicted a higher probability for choice repetition independent of the actor. In addition, the model’s predicted values computed for the interaction $A_{n - 2} \times D_{n - 2 }$ revealed differing effects for own vs. partner. For instance, when own responds right at 2-back, the predicted probability of repeating right is 0.81; however, when partner responds right at 2-back, the probability is lower at 0.77. Similarly, when own responds left at 2-back, the predicted probability of choosing right is 0.72, as compared to when partner responds left at 2-back, which is 0.76. This suggests a choice repetition bias primarily driven by the participant’s own decision at 2-back, even though knowing their action is being observed by the other. The findings align with the literature that sequential perceptual choices depend on the current sensory information and the acting participant’s own trial history responses^8^. However, our results indicate that while the participant shows a choice repetition bias influenced by the decision at 2-back, there is also a tendency to switch responses following their own previous choice. Taken together, the results suggest that choice history is not actor-independent, which only relates to the combined sequence of decisions as stated in the first hypothesis. Instead, the actors and the decisions in history up to 2-back show a differing influence on the current choice.

Overall, our findings extend the existing research on choice history bias in decision-making^7,21^ by investigating it using a shared perceptual task. While we observed a tendency for choice alternation following the participant’s own previous response, which is similar to following the partner’s decision, evidence for a strong dyadic influence is limited. Nonetheless, the significance of the interaction effects of actor and decision at both 1-back and 2-back show support for the alternative hypothesis that the participant acknowledged the partner’s decisions yet did not adhere to them either.

The present study contributes to the broader research work on the role of social interaction in perceptual decision-making^25–27^. Our task design differed from past work on choice history bias by including a second person. We present a model suggesting that perceptual judgment might not be solely individualistic. Future research could further explore dyadic dynamics by examining RTs of choice sequences. For example, investigating whether participants become faster following their own consecutive decisions or slow down following their partner’s consecutive decisions could indicate potential attentional shifts. In addition, future work can extend dyadic interaction to other scenarios. For example, using a perceptual task, a human participant could interact and observe a computer that mimics the participant’s behavior. Specifically, one can examine how choice history bias might emerge or change under three conditions, such as when the computer is consistently correct, consistently wrong, or follows a mixed pattern more similar to naturalistic human decision-making. Given that people typically attribute social qualities to computers^28^, the extent to which there might be a joint social perception remains unclear. The results carry broader implications of trust in technology and adapting choice decisions to external agents. In conclusion, we have explored the choice history bias effect in dyadic perceptual decision-making, which suggests a more realistic approach to understanding cognition as, in reality, humans are not isolated decision-makers.

## Electronic supplementary material

Below is the link to the electronic supplementary material.

Supplementary Material 1

## Data Availability

The code of this project is publicly available on the Open Science Framework: https://osf.io/3v4m8/; Identifier: DOI 10.17605/OSF.IO/3V4M8.
