# Supplementary material for: Choice history biases in dyadic decision making
Source: Sci Rep. 2025 Apr 3;15:11420. doi: 10.1038/s41598-025-96182-5 (PMC11968856; doi:10.1038/s41598-025-96182-5)
Supplement: Supplementary file 1 — Supplementary Material 1 [file 41598_2025_96182_MOESM1_ESM.pdf]

Choice history biases in dyadic decision making

Ann Huang[1], Mathis Pink[1], Viktoria Zemliak[1], Artur Czeszumski[1,2], Peter König[1,3]

[1]Institute of Cognitive Science, University of Osnabrück, Osnabrück, Germany
[2]Social Neuroscience Lab, Institute of Psychology, Polish Academy of Sciences, Warsaw, Poland
[3]Department of Neurophysiology and Pathophysiology, University Medical Center Hamburg-Eppendorf, Hamburg, Germany

Correspondence concerning this article should be addressed to Ann Huang, Institute of Cognitive Science, University of Osnabrück, Wachsbleiche 27, 49090 Osnabrück, Germany. Email: ann.huang@uni-osnabrueck.de. ORCID ID: 0000-0001-8049-7755

SUPPLEMENTARY MATERIALS

Supplementary Methods

We used an adaptive approach to achieve an overall target of 75% mean accuracy. In other words, while the participants were simultaneously presented with stimuli moving in identical directions, the stimuli difficulty level was tailored to each participant based on his/her behavioral data. We implemented such an adaptive procedure because our extensive pilot tests, which used a logistic Weibull function to estimate the individual psychometric curve, showed notable misalignment in the accuracy outcomes between the titration block and the main experiment. The adaptive approach was implemented by fitting a drift-diffusion model (PyDDM) to the mean reaction time (RT) and accuracy data from the titration block of the experiment (Palmer et al., 2005; Shinn et al., 2020). This proportional-rate diffusion model used a maximum likelihood procedure to estimate each participant's psychometric and chronometric functions. The psychometric curve parameterization was derived from the product of the drift rate and decision bound. The drift-diffusion model was re-fitted to the behavioral data after every experiment block to re-align the task difficulty with the participant's behavior during the main experiment. Unlike the logistic Weibull function that only fitted the accuracy data, the drift-diffusion model used more data, including the response, RT, and accuracy, and accounted for the entire titration block. The adaptive procedure enabled task difficulty adjustments throughout the main experiment, thus minimizing large deviations from the targeted accuracy outcomes.

Supplementary Results

1) $(A_{n-1}^{+1} D_{n-1}^{-1})(A_{n-2}^{-1} D_{n-2}^{-1})$, or **own left** decision at 1-back, combined with **partner left** decision at 2-back:
(-0.03)(1) + (0.00)(-1) + (0.11)(1)(-1) + (-0.03)(-1) + (0.08)(-1) + (0.27)(-1)(-1) = **0.08**

2) $(A_{n-1}^{-1} D_{n-1}^{-1})(A_{n-2}^{+1} D_{n-2}^{-1})$, or **partner left** decision at 1-back, combined with **own left** decision at 2-back:
(-0.03)(-1) + (0.00)(-1) + (0.11)(-1)(-1) + (-0.04)(1) + (0.03)(-1) + (0.19)(1)(-1) = **-0.12**

3) $(A_{n-1}^{+1}D_{n-1}^{+1})(A_{n-2}^{-1}D_{n-2}^{+1})$, or **own right** decision at 1-back, combined with **partner right** decision at 2-back:
(-0.03)(1) + (0.00)(1) + (0.11)(1)(1) + (-0.02)(-1) + (0.08)(1) + (0.22)(-1)(1) = **-0.04**

4) $(A_{n-1}^{-1}D_{n-1}^{+1})(A_{n-2}^{+1}D_{n-2}^{+1})$, or **partner right** decision at 1-back, combined with **own right** decision at 2-back:
(-0.03)(-1) + (0.00)(1) + (0.11)(-1)(1) + (-0.05)(1) + (0.02)(1) + (0.14)(1)(1) = **0.03**

5) $(A_{n-1}^{+1}D_{n-1}^{-1})(A_{n-2}^{-1}D_{n-2}^{+1})$, or **own left** decision at 1-back, combined with **partner right** decision at 2-back:
(-0.03)(1) + (0.00)(-1) +(0.11)(+1)(-1) + (-0.03)(-1) + (0.08)(1) + (0.27)(-1)(1) = **-0.30**

6) $(A_{n-1}^{-1}D_{n-1}^{-1})(A_{n-2}^{+1}D_{n-2}^{+1})$, or **partner left** decision at 1-back, combined with **own right** decision at 2-back:
(-0.03)(-1) + (0.00)(-1) + (0.11)(-1)(-1) + (-0.04)(1) + (0.03)(1) + (0.19)(1)(1) = **0.32**

7) $(A_{n-1}^{+1}D_{n-1}^{+1})(A_{n-2}^{-1}D_{n-2}^{-1})$, or **own right** decision at 1-back, combined with **partner left** decision at 2-back:
(-0.03)(1) + (0.00)(1) + (0.11)(1)(1) + (-0.02)(-1) + (0.08)(-1) + (0.22)(-1)(-1) = **0.30**

8) $(A_{n-1}^{-1}D_{n-1}^{+1})(A_{n-2}^{+1}D_{n-2}^{-1})$, or **partner right** decision at 1-back, combined with **own left** decision at 2-back:
(-0.03)(-1) + (0.00)(1) + (0.11)(-1)(1) + (-0.05)(-1) + (0.02)(-1) + (0.14)(1)(-1) = **-0.29**
